# The Leaderless Bacteriocin Enterocin K1 Is Highly Potent against *Enterococcus faecium*: A Study on Structure, Target Spectrum and Receptor

**DOI:** 10.3389/fmicb.2017.00774

**Published:** 2017-05-03

**Authors:** Kirill V. Ovchinnikov, Per Eugen Kristiansen, Daniel Straume, Marianne S. Jensen, Tamara Aleksandrzak-Piekarczyk, Ingolf F. Nes, Dzung B. Diep

**Affiliations:** ^1^Department of Chemistry, Biotechnology and Food Science, Norwegian University of Life SciencesÅs, Norway; ^2^Department of Biosciences, University of OsloOslo, Norway; ^3^Institute of Biochemistry and Biophysics, Polish Academy of SciencesWarsaw, Poland

**Keywords:** leaderless bacteriocins, NMR, receptors, RseP, enterococci

## Abstract

Enterocin K1 (EntK1), enterocin EJ97 (EntEJ97), and LsbB are three sequence related leaderless bacteriocins. Yet LsbB kills only lactococci while EntK1 and EntEJ97 target wider spectra with EntK1 being particularly active against *Enterococcus faecium*, including nosocomial multidrug resistant isolates. NMR study of EntK1 showed that it had a structure very similar to LsbB – both having an amphiphilic N-terminal α-helix and an unstructured C-terminus. The α-helix in EntK1 is, however, about 3–4 residues longer than that of LsbB. Enterococcal mutants highly resistant to EntEJ97 and EntK1 were found to have mutations within *rseP*, a gene encoding a stress response membrane-bound Zn-dependent protease. Heterologous expression of the enterococcal *rseP* rendered resistant cells of *Streptococcus pneumoniae* sensitive to EntK1 and EntEJ97, suggesting that RseP likely serves as the receptor for EntK1 and EntEJ97. It was also shown that the conserved proteolytic active site in *E. faecalis* RseP is partly required for EntK1 and EntEJ97 activity, since alanine substitutions of its conserved residues (HExxH) reduced the sensitivity of the clones to the bacteriocins. RseP is known to be involved in bacterial stress response. As expected, the growth of resistant mutants with mutations within *rseP* was severely affected when they were exposed to higher (stressing) growth temperatures, e.g., at 45°C, at which wild type cells still grew well. These findings allow us to design a hurdle strategy with a combination of the bacteriocin(s) and higher temperature that effectively kills bacteriocin sensitive bacteria and prevents the development of resistant cells.

## Introduction

The spread of antibiotic-resistant bacteria poses a great threat to public health and is growing worse since the current progress in developing of new antibiotics is limited (Brown and Wright, 2016). Aside from the introduction of carbapenems in 1985, all new antibiotics between the early 1960s and 2000 were synthetic derivatives of existing scaffolds, which often allow resistant strains to arise rapidly (Fischbach and Walsh, 2009). Only in the USA the economic loss provoked by antibiotic resistance is estimated to be up to 55 billion USD per year with some data suggesting that the total cost can be even higher (Smith and Coast, 2013). Consequently, there is a need for new antimicrobials that can be used as alternatives to conventional antibiotics. Among such alternatives the antimicrobial peptides, the bacteriocins, are potential antibacterial agents to fight infections (Cotter et al., 2013).

Bacteriocins are ribosomally synthesized peptides produced by bacteria to inhibit or kill other bacteria in competition for nutrients or habitats. They are almost with no exception cationic, amphiphilic, and heat-stable. The antimicrobial inhibition spectra and potency (strength) of bacteriocins differ greatly, with the majority being active only against closely related bacteria while some being much broader, targeting cells from different genera (Nes et al., 2014).

Bacteriocins have a number of advantages over conventional antibiotics and chemical food preservatives. Bacteriocins show antimicrobial activity at pico- to nanomolar concentrations and are commonly produced by bacteria found in many fermented foods and in the human gut microflora – properties making them very attractive as natural food preservatives (Nes et al., 1996). Most bacteriocins from gram positive bacteria are known as membrane-active peptides, i.e., they disrupt membrane integrity, leading to leakage of intracellular solutes and cell death (Kjos et al., 2011). This killing mechanism is different from that of most antibiotics, which often act as enzyme inhibitors of key metabolic pathways. Due to their different modes of action, bacteriocins do not discriminate between antibiotic resistant and sensitive bacteria (Cotter et al., 2013).

Most bacteriocins are synthesized as a propeptide with an N-terminal leader which is removed by a dedicated ABC transporter or the Sec system during exporting from cells (Havarstein et al., 1995; Cintas et al., 1997). However, there is a group of so-called leaderless bacteriocins, which are synthesized without an N-terminal leader and the mechanism behind their export is unknown. Leaderless bacteriocins are often relatively small (30–50 amino acids) and contain no modifications. These features make them relatively easy to obtain by synthetic means, thereby opening new opportunities for detailed bacteriocin study and design of new peptides with improved properties (Ovchinnikov et al., 2014).

Enterocin K1 (EntK1) is a leaderless bacteriocin, which belongs to LsbB bacteriocin family. The family presently contains four members: LsbB, EntK1, EntQ, and EntEJ97 (Ovchinnikov et al., 2014), all being leaderless and non-modified peptides. LsbB is a 30 amino acid (aa) residues peptide produced by *Lactococcus lactis.* It has a very narrow inhibition spectrum which contains only lactococcal strains (Gajic et al., 2003). The remaining three bacteriocins are produced by different enterococcal strains. EntQ (34 aa residues) and especially EntEJ97 (44 aa residues) have broader antimicrobial spectra than LsbB (Galvez et al., 1998; Cintas et al., 2000; Basanta et al., 2008). Less is known about the activity of the newly discovered EntK1 (37 aa residues), although it has been shown to inhibit *L. lactis* IL1403 (Ovchinnikov et al., 2014).

It is has been shown for an increasing number of bacteriocins that they kill target cells in a receptor-mediated manner, i.e., bacteriocins recognize specific receptors on target cells where they bind and cause membrane-disruption that eventually leads to cell death (Diep et al., 2007; Cotter, 2014). Recently, it has been shown that the lactococcal Zn-dependent metallopeptidase YvjB (also known as RseP) belonging to site-2 protease (S2P) protein family, serves as the receptor for LsbB (Uzelac et al., 2013; Miljkovic et al., 2016). Our previous structure-function study of LsbB provided strong evidence that the peptide binds to its receptor using its unstructured C-terminal part (Ovchinnikov et al., 2014).

In this work we determined the structure of EntK1, its activity spectrum and unravel the details behind target specificity of EntK1 and its sequence related bacteriocin EntEJ97. Based on the fact that RseP is involved in bacterial stress response (Varahan et al., 2013) we developed an efficient strategy to improve the activity of EntK1 and its sequence related bacteriocins. We also showed that at least in *Enterococcus faecalis*, another membrane protein is responsible for bacterial sensitivity to EntEJ97, besides EntEJ97 receptor RseP.

## Materials and Methods

### Bacterial Strains, Growth Conditions, Bacteriocins and Antimicrobial Assays

All the strains (see below) used in minimal inhibitory concentration (MIC) assays were grown in BHI medium (Oxoid) at 30°C without shaking. LsbB, EntK1, EntEJ97, and BHT-B bacteriocins were synthesized by Pepmic Co., Ltd, China with 98–99% purity. Synthesized peptides were solubilized to the concentrations of 10.0–0.1 mg/ml in 0.1% (vol/vol) trifluoroacetic acid and stored at -20°C until use. Bacteriocin garvicin ML was purified to 95% as described by (Borrero et al., 2011). Bacteriocin activity was determined by microtiter plate assay as previously described (Holo et al., 1991). The MIC was defined as the minimal bacteriocin concentration that inhibited the growth of the indicator strain by at least 50% (50% of the turbidity of the control culture without bacteriocin) in 200 μl culture.

### CD Spectroscopy

Circular dichroism (CD) spectra were recorded using a Jasco J-810 spectropolarimeter (Jasco International Co.) calibrated with D-camphor-10-sulfonate (Icatayama Chemical). All measurements were done using a quartz cuvette (Starna) with 0.1 cm path length at 25°C. Samples were scanned five times with a scanning rate of 50 nm/min with a bandwidth of 0.5 nm and response time of 1 s over the wavelength range 190–250 nm. Spectra were recorded at different (30 and 50%) trifluoroethanol (TFE) (Aldridge) concentrations at 25°C. The approximate α-helical content of the protein was estimated from its molar ellipticity at 222 nm (Scholtz et al., 1991).

### NMR Spectroscopy

The experiments were run on a sample containing 1.0 mM EntK1, 50% D3-TFE (99.5% D) (Aldrich), Milli-Q water and 0.2 mM of 4,4-dimethyl-4-silapentane-1-sulfonic acid (DSS) (Larodan).

2D NOESY (Jeener et al., 1979), 2D TOCSY (Braunschweiler and Ernst, 1983), 2D DQCOSY, ^15^N-HSQC (Davis et al., 1992), and^13^C-HSQC (Hurd, 1991) were recorded. The data was acquired on a 600 MHz Bruker Avance II spectrometer with four channels and a 5 mm TCI cryoprobe (Bruker Biospin). NOESY spectra with mixing times between 120 and 300 ms were obtained for both samples. TOCSY mixing times of 15–80 ms was used. The experiments were run at 298.15 K. Spectra were processed using the Topspin program (Bruker Biospin). DSS was used as a chemical shift standard, and ^15^N and ^13^C data were referenced using frequency ratios as described in (Wishart et al., 1995).

For visualization, assignment and integration of the spectra the computer program CARA was used (Keller, 2004). The spectra were assigned using standard methods (Wüthrich, 1986).

Dihedral angle restraints were obtained from the chemical shift values using the program TALOS-N (Shen and Bax, 2013). Nuclear Overhauser effect (NOE) distance restraints were calculated from the peak volumes in the NOESY spectra with NOESY mixing time 200 ms.

All structure calculations were made using the structure calculation program CYANA 2.1 (Guntert et al., 1997; Herrmann et al., 2002). The applied restraints are presented in **Table 1**. The annealing macro in CYANA calculated 100 structures. The 20 structures with the lowest energy were kept and analyzed further. The root-mean-square deviation (RMSD) was calculated and the structures were visualized using MolMol (Koradi et al., 1996).

**Table 1:** Structure data and characteristics of the obtained NMR structures.

	EntK1 in 50% TFE
Distance constraints	561
Intra residue	162
Sequential (ji - jj = 1)	176
Medium range (1 < ji - jj < 5)	158
Long range (ji - jj > 5)	24
Dihedral angle constraints from TALOS-N	68
Energies (Å^2^)§	2.06 ± 0.01
Violations^∗^	0
Deviations from idealized geometry	
Bonds (Å)	7.4 ± 0 × 10^-3^
Angles (°)	1.1 ± 0.01
Ramachandran plot	
% in favored regions	77.7
% in allowed regions	22.3
Mean pairwise RMSD	
Backbone/Heavy atoms	0.49 ± 0.19/1.11 ± 0.24
Backbone (8–24)/Heavy atoms (8–24)	0.01 ± 0.00/0.52 ± 0.15

*§CYANA energy. ^∗^ Only violations larger than 0.2 Å or 5°*.

### Generation of Bacteriocin Resistant Mutants of *E. faecalis* and *E. faecium*

EntEJ97 and EntK1-resistant mutants were obtained by spot-on-lawn assay using 20 μl of bacteriocin with concentration 1.0–0.1 mg/ml. After overnight incubation at 30°C, resistant colonies of *E. faecalis* LMG3358 (resistant to EntEJ97) and *E. faecium* LMG2787 (resistant to EntEJ97 and EntK1) appeared within the inhibition zones. Resistant isolates were picked and re-streaked on plates for pure cultures before frozen cultures containing 15% glycerol were made and stored at -80°C. The level of resistance against EntEJ97 and EntK1 was determined by a microtitre plate assay (Holo et al., 1991). DNA from the bacteriocin resistant mutants was extracted from overnight cultures with a GenElute^TM^ Bacterial Genomic DNA Kit (Sigma–Aldrich) and PCR of the *rseP* gene was performed using primers Ent, EF (F, M, R) (**Table 2**). For sequencing of *E. faecium* LMG2787 mutants with transposons inside *rseP*, additional primers (EF_R2, T1_F, T2_F, and T3_F) were created (**Table 2**). PCR products were purified with NucleoSpin Extract II (Macherey-Nagel, Düren, Germany) and sent to GATC Biotech, Germany, for sequencing.

**Table 2:** Primers used in the study.

Primer	Oligonucleotide sequence (5′?3′)	Reference
Ent_F	CGAAGTGGTCAAGTCCAATGGT	This study
Ent_M	GTGCGGATTGCGCCACTTGAC	This study
Ent_R	GATGACTTAAGACTTCTGCATCAT	This study
EF_F	GCTCTTAGCAAGATTTGATGGC	This study
EF_M	CGTCCACACTGACTACCTCATC	This study
EF_R	CTTAGACCGTTTCGACAGTTTGC	This study
EF_R2	TGCAATCTGTCGACGTGACAC	This study
T1_F	AGCTAGCTCAAAGGAAGAGGC	This study
T2_F	TGCAATCTGTCGACGTGACAC	This study
T3_F	GCTCGAACAGCTAAGAATGCCT	This study
th009	ACGTTTGAGCAATTTCCTTCC	This study
th010	CACATTATCCATTAAAAATCAAACAGCGTTTCCTCCGTCTTTTG	This study
th011	GTCCAAAAGCATAAGGAAAGTCGAGGAATATTATGAAACAAAG	This study
th012	CATTTCCAACTAGAAGGGCTG	This study
ds171	ATTTATATTTATTATTGGAGGTTCAATGAAAACAATTATCACATTCA	This study
ds172	ATTGGGAAGAGTTACATATTAGAAATTAAAAGAAAAAGCGTTGAATATC	This study
ds87	AGCGTTTCCTCCGTCTTTTG	This study
ds88	CAAAAGACGGAGGAAACGCTTCGAGGAATATTATGAAACAAAG	This study
Kan484.F	GTTTGATTTTTAATGGATAATGTG	Johnsborg et al., 2008
RpsL41.R	CTTTCCTTATGCTTTTGGAC	Johnsborg et al., 2008
khb31	ATAACAAATCCAGTAGCTTTGG	Berg et al., 2011
khb33	TTTCTAATATGTAACTCTTCCCAAT	Berg et al., 2011
khb34	CATCGGAACCTATACTCTTTTAG	Berg et al., 2011
khb36	TGAACCTCCAATAATAAATATAAAT	Berg et al., 2011
466p1	GGTATTCTTGTCCTCGTAGCTGAATTTGGCCACTTTTATTTTGC	This study
467p2	GCAAAATAAAAGTGGCCAAATTCAGCTACGAGGACAAGAATACC	This study
468p1	ATTCTTGTCCTCGTACATGCATTTGGCCACTTTTATTTTGCAAAAC	This study
469p2	GTTTTGCAAAATAAAAGTGGCCAAATGCATGTACGAGGACAAGAAT	This study
470p1	GTACATGAATTTGGCGCTTTTTATTTTGCAAAACGAGC	This study
471p2	GCTCGTTTTGCAAAATAAAAAGCGCCAAATTCATGTAC	This study
472-p1	GAATTTGGCCACTTTGCTTTTGCAAAACGAGC	This study
473-p2	GCTCGTTTTGCAAAAGCAAAGTGGCCAAATTC	This study
474p1	ATTCTTGTCCTCGTAGCTGCATTTGGCGCCTTTTATTTTGCAAAACGAGC	This study
475p2	GCTCGTTTTGCAAAATAAAAGGCGCCAAATGCAGCTACGAGGACAAGAAT	This study

### Construction of *S. pneumoniae* Transformants and Mutants of *rseP*

To express the *E. faecalis rseP* gene heterologously in *S. pneumoniae*, the gene was placed by homologous recombination in the genome of strain SPH131, behind the ComS-inducible P*_comX_* promoter (ComRS system) (Berg et al., 2011). The P*_comX_*-*rseP* construct was created by overlap extension PCR (Higuchi et al., 1988). First, the *E. faecalis* LMG3358 *rseP* was amplified using the primer pair ds171/ds172 with genomic *E. faecalis* DNA as template. The P*_comX_* promoter and its ∼1000 bp upstream and downstream regions were amplified using the primer pairs khb31/khb36 and khb33/khb34, respectively. Genomic DNA derived from strain SPH131 served as template. The P*_comX_* with its upstream region was fused to the 5′ end of the *E. faecalis rseP* gene using the primers khb31 and ds172. The P*_comX_* downstream fragment was fused to the 3′ end of the *rseP* gene using primer pair ds171 and khb34. Finally, these two fragments were fused using primer khb31 and khb34 giving rise to P*_comX_*-*rseP*. The Janus cassette (Sung et al., 2001) in strain SPH131 was replaced with the P*_comX_*-*rseP* fragment by natural transformation, giving rise to strain ds218 (**Table 3** and Supplementary Figure 1).

**Table 3:** *Streptococcus pneumoniae* strains used in the study.

Strain	Genotype/relevant features^a^	Reference/source
RH1	*S. pneumoniae*, R704, but *ebg*::*spc*; Ery^r^ Spc^r^	Johnsborg et al., 2008
RH426	*S. pneumoniae*, contains the Janus cassette; Ery^r^ Kan^r^	Johnsborg and Håvarstein, 2009
SPH131	*S. pneumoniae*, contains the ComRS system, Janus cassette is placed behind P*_comX_*; Ery^r^ Kan^r^	Berg et al., 2011
ds218	*S. pneumoniae* SPH131 but Δjanus:: *rseP* from *E. faecalis* LMG5833	This study
ds219	*S. pneumoniae* ds218 but Δ*rseP*_wt_::janus	This study
ds220	*S. pneumoniae* ds219 but Δjanus	This study
ds221	*S. pneumoniae* ds220 but Δ *rseP*::janus	This study
ms1	*S. pneumoniae* ds221 but Δjanus::*EF-rseP-H18 > A*	This study
ms2	*S. pneumoniae* ds221 but Δjanus::*EF-rseP-E19 > A*	This study
ms3	*S. pneumoniae* ds221 but Δjanus::*EF-rseP-H22 > A*	This study
ms4	*S. pneumoniae* ds221 but Δjanus::*EF-rseP-Y24 > A*	This study
ms5	*S. pneumoniae* ds221 but Δjanus::*EF-rseP-HExxH > AAxxA*	This study

^a^*Cm, chloramphenicol; Ery, erythromycin; Spc, spectinomycin; Kan, kanamycin; Sm, streptomycin*.

*Streptococcus pneumoniae* has a gene homologous to the lactococcal *rseP*. To avoid the potential background noise of the *S. pneumoniae rseP*, this gene was removed from the genome in strain ds218 using the Janus cassette (Sung et al., 2001). An *rseP* deletion cassette was constructed by amplifying Janus with the primers kan484F and RpsL41.R (Johnsborg et al., 2008) where genomic DNA from strain RH426 (Johnsborg et al., 2008) served as template. The Janus cassette was then fused to the ∼1000 bp upstream [primers th009 and th010 with genomic RH1 (Johnsborg et al., 2008) DNA as template] and downstream region (primers th011 and th012 with genomic RH1 DNA as template) of *rseP* using primers th009 and th012. The resulting fragment was used to transform strain ds218 resulting in the replacement of *S. pneumoniae rseP* with Janus generating strain ds219. Janus was then removed from strain ds219 by transforming with a fragment consisting of the *rseP* flanking regions only. This fragment was constructed by amplifying the *rseP* ∼1000 bp upstream region using primers th009 and ds87, while the ∼1000 bp downstream region was amplified with the primers ds88 and th012. The upstream and downstream fragments where then fused using the primers pair th009/th012. The resulting fragment was used to replace the Janus in strain ds219 resulting in strain ds220 (**Table 3** and Supplementary Figure 1).

To create a strain expressing point mutated *rseP* genes we replaced the *E. faecalis* 3358 *rseP* in strain ds220 with Janus cassette giving rise to strain ds221. This Janus cassette was amplified with the primer pair khb31/khb34 and genomic DNA from strain SPH131 served as template. Selected residues in RseP were substituted with alanines by a PCR approach, using primer pairs listed in **Table 2**. The resulting DNA pair fragments were subsequently fused using the primers khb31 and khb34. The resulting fragment was used to replace Janus cassette in strain ds221 giving rise to strains ms1-5 (**Table 3**). Ectopic expression of the *rseP* gene in strain ds220 and ms1-5 was induced with the synthetic ComS peptide (NH2-LPYFAGCL-COOH) (Genosphere Biotechnologies) as described by Berg et al. (2011).

## Results

### Structural Analysis of EntK1 by CD and NMR-Spectroscopy

Circular dichroism spectra of EntK1 showed that it was unstructured in water but became structured in TFE solution (Supplementary Figure 2). Maximum structuring was obtained in 50% TFE (55% α-helical content). This concentration was consequently used in the NMR experiment.

**FIGURE 1:**
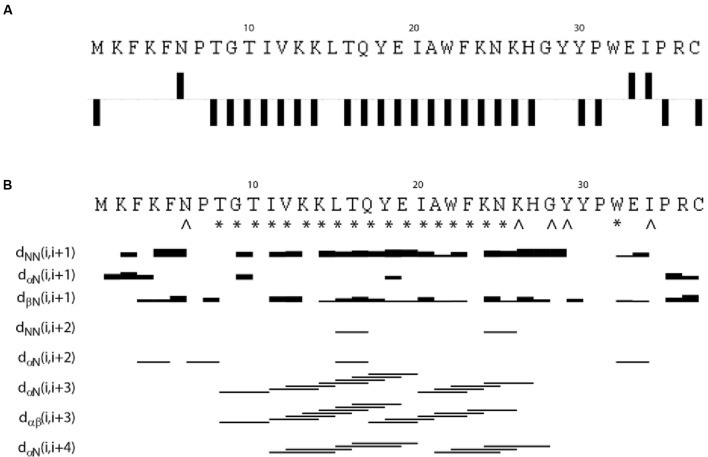
**Results from analysis of NMR chemical shifts and nuclear Overhauser effects (NOE’s) of EntK1 in TFE**. Chemical shift index (CSI) of Hα carbons is shown in **(A)**. Intermediate NOE restraints and residues where determined torsion angles from the chemical shifts are shown in **(B)**. The signs ^∗^ and ˆ indicate that TALOS determined phi and psi torsion angle restraints, the ^∗^ are used to symbolize torsion angle restraints in the α-helical region of the Ramachandran plot while ˆ is used for all other torsion angles. The lines indicate typical α-helical restraints obtained from analysis of the NOE spectra. Thicknesses of the lines shown are related to NOE cross peak intensities.

The NMR spectra were assigned using standard methods as described in “Materials and Methods.” Supplementary Figure 3 shows the assigned ^15^N HSQC spectrum of EntK1 in 50% TFE. **Figure 1** shows connected strips from NOESY, the torsion angle restraints used in structure calculations and the Hα chemical shift indexes (CSIs). CSI (low Hα ppm values) (Matthews et al., 1992) indicates that there is an α-helix in EntK1 from residue 8 to 27 and TALOS-N torsion angle predictions (Shen and Bax, 2013) indicated backbone torsion angles (ϕ- and ψ-angle) consistent with α-helical regions from residue 8 to 25 (**Figure 1**).

A total of 561 (15.1 per residue) unique NOE connectivities were assigned. The total number of distance restrictions and their classes are shown in **Table 1**. The mid-range NOE connectivities that are especially important for α-helices, NN(i, i+2), αN(i, i+2), αN(i, i+3), αβ(i, i+3), and αN(i, i+4), are shown in **Figure 1**. In the presence of TFE, the observed connectivities indicated an α-helical region stretching from residue 8 to 26.

**FIGURE 2:**
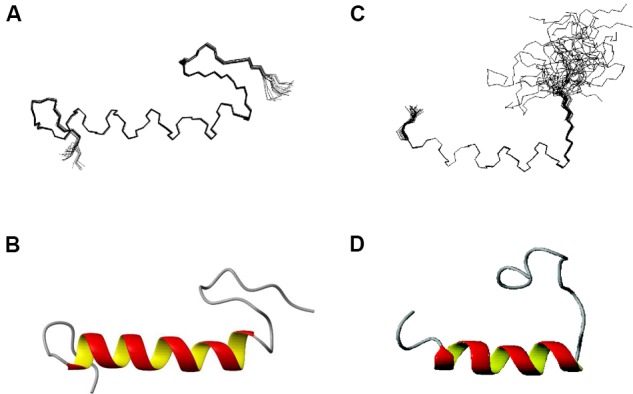
**NMR structure of EntK1 **(A,B)** compared with LsbB **(C,D)** (Ovchinnikov et al., 2014) in 50% TFE**. The structures ensembles of the 20 lowest energy structures superimposed are shown in **(A,C)** and cartoon representations of the lowest energy structures are shown in **(B,D)**.

Structures of EntK1 based on the experimentally obtained constrains were calculated using CYANA. A superimposition of the structure ensemble of the 20 lowest energy structures of EntK1 and the cartoon depiction of the lowest energy structure of EntK1 are shown in **Figures 2A,B**, respectively. The NMR structure of EntK1 contains an α-helix from residue 8 to 24. The structure ensemble has been deposited to the Protein Data Bank with access code: 5L82 and the NMR data have been deposited to the Biomagnetic Resonance Data Bank with the access code 34006.

### The Inhibitory Spectra of the EntK1 and Two Sequence Related Bacteriocins

A sizable collection of Gram-positive bacteria from different species and genera was used as indicators to assess the inhibition spectra of the bacteriocins EntK1, EntEJ97, and LsbB (**Table 4**). EntQ, which is a member of the LsbB family bacteriocins, was not tested because synthetic EntQ peptides had poor activity, likely due to formation of disulphide bridges between and inside the molecules, since adding reducing agents (e.g., DTT) restored some of the activity of EntQ (data not shown). As seen in **Table 4**, the antimicrobial spectra of EntK1, EntEJ97, and LsbB differ greatly. LsbB was active only against *L. lactis* IL1403, one of the four strains of *L. lactis* tested and it had no activity against other genera and species. EntK1 was active mostly against *E. faecium* and *E. hirae* and some lactococcal strains. EntEJ97 showed the broadest activity spectrum, inhibiting *E. faecium, E. faecalis, L. lactis, Staphylococcus aureus, Lactococcus garvieae*, and *Listeria monocytogenes*. It is noteworthy that EntK1 appeared to have significantly lower MIC values (i.e., being more potent) than EntEJ97 against *E. faecium*, but was inactive against *E. faecalis* (**Table 4**). Similarly, all three bacteriocins were active against the *L. lactis* IL1403, but LsbB was about 340 and 70 times more potent than EntK1 and EntEJ97, respectively (**Table 4**).

**Table 4:** Minimal inhibitory concentration (MIC) values (nM) of EntK1 and its related bacteriocins against different bacterial species.

Indicator strain^∗^	LsbB	EntK1	Ent-EJ97
*Staphylococcus aureus* (*n* = 6)	>7000	>5500	590- >4500
*Staphylococcus epidermidis* (*n* = 1)	>7000	>5500	>4700
*Enterococcus faecalis* (*n* = 11)	>7000	2700- > 5500	145–295
*Enterococcus faecium* (*n* = 8)	>7000	10–85	145–295
*Enterococcus faecium* (*n* = 10)^∗∗^	>7000	10–45	75–145
*Enterococcus hirae* (*n* = 1)	>7000	45	75
*Lactococcus garvieae* (*n* = 2)	>7000	340–1370	295
*Lactobacillus* spp. (*n* = 3)	>7000	5500- >5500	145- >4700
*Lactococcus lactis* IL1403	0.5	170	37
*Lactococcus lactis* (*n* = 3)	>7000	>5500	145–295
*Bacillus cereus* (*n* = 5)	>7000	2750- >5500	2300- >4700
*Listeria monocytogenes* (*n* = 2)	>7000	>5500	1175–2300

*^∗^The numbers in parentheses are referred to the number of strains tested. ^∗∗^Nosocomial isolates from different European hospitals, including VRE*.

### Different Levels of Resistance against EntK1 and EntEJ97 in *E. faecalis* and *E. faecium*

The strain *E. faecium* LMG2787 was used to generate mutants resistant to EntK1 and EntEJ97. Since *E. faecalis* is resistant to EntK1, *E. faecalis* LMG3358 was used to generate mutants resistant only to EntEJ97. The two enterococcal strains were exposed to various concentrations of EntEJ97 and EntK1 at 30°C and relatively many resistant colonies appeared within the inhibition zones on agar plates (see below). For *E. faecalis*, 12 EntEJ97 resistant colonies were randomly selected and examined for resistance to EntEJ97 by microtiter assays. Similarly, six EntEJ97 and six EntK1mutants of *E. faecium* were selected for further analysis.

Two levels of *E. faecalis* resistance to EntEJ97 were found: seven colonies were highly resistant (HR) – at least 500 times more compared to the wild type (WT) strain. The second set of five colonies showed a lower resistance (LR) level, being about 16–32 times more resistant than the WT *E. faecalis*. For *E. faecium* only HR (at least 500 times against EntK1 and EntEJ97, compared to WT) type mutants were found. When all these mutants were challenged with the non-related bacteriocins BHT-B (Hyink et al., 2005) and garvicin ML (Borrero et al., 2011), they were found as sensitive as the WT strains (data not shown). These results clearly imply the involvement of specific resistance mechanism(s) toward EntEJ97/EntK1 amongst these mutants.

### Highly Resistant Mutants of *E. faecalis* and *E. faecium* Have Mutations in the *rseP* Gene

**FIGURE 3:**
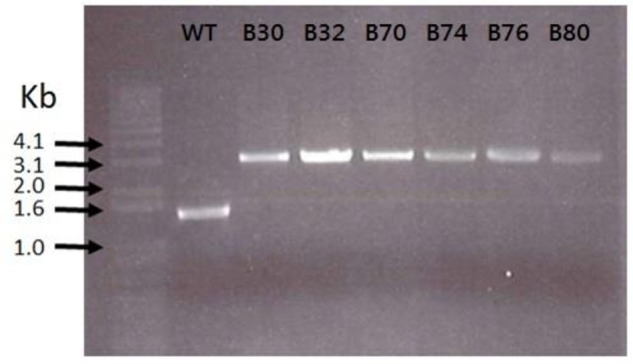
**Transposon insertions in *E. faecium* LMG2787 *rseP* mutants (B30–B80) compared to the wild type (WT)**.

As the lactococcal RseP has previously been found to serve as receptor for LsbB, we suspected that the RseP homologous in *Enterococcus* might serve the same function for the LsbB sequence related EntEJ97/EntK1 bacteriocins. The DNA regions containing *rseP* in all the EntEJ97/EntK1-resistant mutants (high and low) were therefore obtained by PCR and sequenced. Different types of mutations were found. For *E. faecalis*, all seven HR mutants contained either one or three consecutive 8-bp repeats of the sequence CAAAAAAT within the *rseP* gene, while the WT *rseP* has two such repeats. On the other hand, all *E. faecium* HR mutants carried a transposon within *rseP* (**Figure 3**). In all cases, these mutations caused frameshift in *rseP* and premature termination, indicating that a functional ResP is necessary for the sensitivity toward EntEJ97/EntK1. Surprisingly, no mutations were found in *rseP* from all five EntEJ97 LR mutants of *E. faecalis*, indicating that additional genes can affect the sensitivity of the bacterium to these bacteriocins. This latter aspect will be further mentioned in the “Discussion” section.

### *rseP* Expression in *S. pneumoniae* Confers Sensitivity to EntEJ97/EntK1

In order to confirm that the expression of *rseP* is sufficient to confer sensitivity to EntEJ97 and EntK1, the *rseP* gene from *E. faecalis* LMG3358 was heterologously expressed in the distantly related and non-sensitive host *S. pneumoniae* strain SPH131 as described in (Berg et al., 2011). To avoid potential background noise the endogenous *rseP* of *S. pneumoniae* SPH131 was removed by homologous recombination, giving rise to the clone *S. pneumoniae* ds220 with the endogenous *rseP* deleted but with the enterococcal *rseP* expressed under the control of the inducible promoter P*_comX_* (Berg et al., 2011). While non-induced cells were not sensitive to EntEJ97 and EntK1, they became sensitive to the bacteriocins upon induction of *rseP* (**Table 5**). The results indicate that the enterococcal *rseP* is indeed directly involved in the sensitivity to EntEJ97 and EntK1. Further, it should be noted that the induced clone (expressing the *E. faecalis rseP*) was most sensitive to EntEJ97 (MIC of 18 nM), intermediate-sensitive to EntK1 (MIC of 700 nM), and poorly sensitive to LsbB (MIC > 7000 nM). This observation is line with the results presented in **Table 4**, where the *E. faecalis* strains showed a corresponding differentiation in sensitivity to the three bacteriocins.

**Table 5:** Sensitivity of *S. pneumonia* clones to EntEJ97, EntK1, and LsbB.

Strain	Mutation	MIC (nM)^∗^		
		EntEJ97	EntK1	LsbB
’ ds220 non-induced	WT *rseP*	>4500	>5500	>7000
ds220 induced	WT *rseP*	18	700	>7000
ms1	H18 > A	300	>5500	>7000
ms2	E19 > A	300	>5500	>7000
ms3	H22 > A	300	>5500	>7000
ms4	Y24 > A	18	700	>7000
ms5	HExxH > AAxxA	600	>5500	>7000
ds 221	no *rseP*	>4500	>5500	>7000

*^∗^Bacteriocins were active only when *rseP* was induced with ComS (2 μM). Without ComS the cells were resistant to the bacteriocins. ComS was not toxic to the cells even at 20 μM*.

### Mutations within the Active Site of RseP Reduce Sensitivity to EntEJ97/EntK1

Members of the RseP protein family have a conserved proteolytic motif (HExxH, where x is any amino acid) located within the first transmembrane helix (Koide et al., 2007). To analyze whether this active site is important for bacteriocin sensitivity, we changed the invariant residues in this motif (i.e., H18, E19, and H22), one by one, and three altogether in the enterococcal RseP to alanine and then assessed for bacteriocin sensitivity. In addition, the non-conserved residue Y24 located nearby the active site was replaced with alanine to serve as a control. The result showed that all clones expressing the RseP protein in which conserved residues were changed to alanine became less sensitive to EntEJ97 and EntK1, especially when all three conserved residues were replaced with alanine (30 times less sensitive to EntEJ97 than the WT) (**Table 5**). In the control clone (Y24 > A) no changes in the bacteriocin sensitivity were observed. This result demonstrates that the active site of RseP is essential for bacteriocin activity.

### The Growth of EntK1/EntEJ97 Resistant Mutants Is Prevented at Elevated Growth Temperature

**FIGURE 4:**
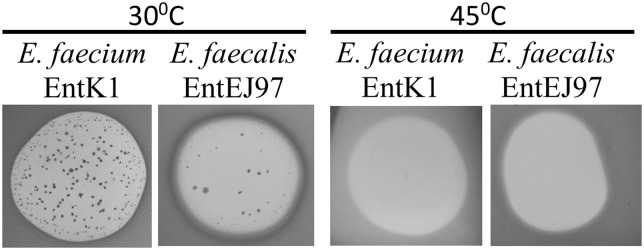
**EntK1 and EntEJ97 resistant mutants appear on at 30°C but not at the elevated temperature 45°C**. Bacteriocin (20 μl of 1 mg/ml) was applied to soft agar containing indicator cells on plates, which was then incubated overnight at indicated temperatures for growth and developing the inhibition zones and resistant colonies.

RseP protein is known to play a key role in bacterial stress response in *Escherichia coli*, *Bacillus subtilis*, and other bacteria (Alba et al., 2002; Varahan et al., 2013; Kim, 2015). We therefore examined how elevated growth temperature as a stress factor, influences the development of EntEJ97 and EntK1 resistance in *E. faecium* and *E. faecalis*. As expected resistant mutants only appeared at 30°C but not at 45°C (**Figure 4**). Cell counts of WT cultures grown for 8 h at 30 and 45°C in BHI broth showed that the cell number at 30°C was only about 1.2 times higher than that at 45°C (data not shown), implying that the lack of resistant mutants at the elevated temperature was not due to poor growth of WT cells but rather due to lack of development of resistant mutants. This is further supported by the fact that EntEJ97/EntK1-resistant mutants with a non-functional *rseP* gene (described above) obtained at 30°C could not grow at 45°C, while WT cells grew well (data not shown).

## Discussion

Structural analyses by CD spectroscopy showed that EntK1 was unstructured in water but became structured when exposed to membrane-mimicking environments (DPC-micelles or TFE). This is a common property for the majority of bacteriocins (Montalban-Lopez et al., 2008; Martin-Visscher et al., 2009).

The structure of EntK1 in 50% TFE is very similar to that of LsbB (**Figure 2**). Both bacteriocins have an N-terminal part mostly composed of an amphiphilic α-helical motif, and an unstructured C-terminal half. EntK1 α-helix is longer than the one of LsbB, being 16–19 residues, versus 13–15 residues in LsbB. A closer look at the α-helices of EntK1 and LsbB showed that both are amphiphilic with the basic amino acids along one side of the helix and non-polar residues along the other side (data not shown). Such amphiphilic distribution is common for many bacteriocins as well as other antimicrobial peptides (Kristiansen et al., 2005; Rogne et al., 2008; Last and Miranker, 2013). The α-helical part of the bacteriocins is probably involved in pore-formation, where the hydrophobic part is facing the hydrophobic membrane or a membrane-located protein (receptor) while the hydrophilic part is facing the inner part of the pore to cause cellular leakage (Kjos et al., 2011).

The C-terminal unstructured parts of EntK1 and LsbB are about the same lengths, 10–13 residues and more similar in sequence. Structure prediction servers (PONDR^®^VL-XT, JPred 4) indicated that EntEJ97 also contains an α-helix from Lys10 to Gly 35 with an unstructured C-terminal tail of 10 residues, which corresponds with our data on LsbB and EntK1 structures.

Besides EntK1 and LsbB, structures of three other leaderless bacteriocins have been obtained so far (enterocin 7, lacticin Q, and aureocin A53). Enterocin 7 is a two-peptide bacteriocin, where the two peptides constituting the bacteriocin are related in sequence and both have a similar fold, consisting of N-terminal, middle and C-terminal helices (Lohans et al., 2013). Lacticin Q and aureocin A53 both consist of four helices surrounding a hydrophobic core (Acedo et al., 2016). The structures of enterocin 7, lacticin Q, and aureocin A53 resemble the structure of circular bacteriocins, and all of them are highly structured in aqueous solutions. As such, EntK1 and LsbB are very different from these bacteriocins by being smaller (30 and 37 residues for LsbB and K1 versus 43–53 residues for the others), unstructured in water (partly due to the absence of any modifications such as disulphide bridges or circularization in these leaderless bacteriocins) and containing only one helix (**Figure 2**).

The lactococcal Zn-dependent metallopeptidase RseP has been shown to be the receptor for LsbB in *L. lactis* IL1403 (Uzelac et al., 2013; Miljkovic et al., 2016). Our present study provides strong evidence that EntEJ97 and EntK1 employ the homologous RseP in enterococci as receptor. This is based on the fact that mutants of *E. faecalis* LMG3358 and *E. faecium* LMG2787 that are HR to EntEJ97/EntK1, contain frameshift mutations in *rseP* and, more conclusively, heterologous expression of the enterococcal *rseP* rendered resistant pneumococcal cells sensitive to these bacteriocins (**Table 5**). However, at least in the case of *E. faecalis* LMG3358, we also found some EntEJ97-resistant mutants, which were less resistant, compared to those with mutated *rseP* and had intact *rseP*, suggesting that these probably had mutations in other gene(s), see discussion below. Nevertheless, both types of resistance were specific to EntEJ97 and EntK1 because all resistant cells were sensitive to the non-related bacteriocins BHT-B (leaderless type) and garvicin ML (circular type). Furthermore, both (high and low) types of resistance share one common feature, that they emerged at the standard growth temperature 30°C but not at the elevated 45°C, indicating that both resistance types are linked to stress response (**Figure 4**). Indeed, RseP has been shown to be involved in stress response in some bacteria. In *Escherichia coli*, cell envelope stress response leads to proteolytic cleavage of the membrane-bound anti-sigma protein RseA by the RseP protease that eventually leads to σE release in the cytoplasm to transcribe stress response genes (Kroos and Akiyama, 2013; Hizukuri et al., 2014; Akiyama et al., 2015). Similarly, in *E. faecalis*, RseP (also known as Eep) is involved in the stress response via degradation of the anti-sigma protein RsiV. This degradation leads to activation of SigV – enterococcal extracytoplasmic sigma protein that plays a key role in stress response (Varahan et al., 2013). Furthermore, RseP deletion mutants have shown increased susceptibility toward heat, ethanol, lysozyme, and acid compared to the susceptibility of the WT strain (Varahan et al., 2013).

Interestingly, RseP of *E. faecalis* is also involved in a proteolysis of the sex pheromone precursor peptide to form an active pheromone (cCF10) which is released by a cognate ABC transporter, called PptAB. It belongs to the EcsAB family and is composed of two proteins – PptA and PptB (Varahan et al., 2014). It has also been shown in *Bacillus subtilis* that mutations within EcsAB blocked the activity of RasP (*Bacillus* homolog of enterococcal RseP) and made the cells sensitive to stress (Heinrich et al., 2008). Remarkably, when we did whole genome sequencing of the five *E. faecalis* LR mutants with intact *rseP* gene, we discovered frequent mutations within genes *pptA* and *pptB* (preliminary results). Moreover, in EntEJ97 LR mutants of *Pediococcus acidilactici* and *Lactococcus garvieae*, all with an intact *rseP* gene also had mutations in *ecsA* and *ecsB* (data not shown).

Thus, our results also revealed a functional connection between these two systems: RseP and EcsAB, that both are involved in the sensitivity to our bacteriocins. As mentioned elsewhere, YvjB (a homolog of RseP) serves as receptor for LsbB which binds directly to the receptor upon killing target cells (Miljkovic et al., 2016). At present it is not clear the exact role of EcsAB in the sensitivity to bacteriocins. Whether it is directly involved as a receptor as described for RseP or in regulation of RseP function or gene expression awaits further investigation.

Members of the RseP protein family are membrane-bound proteins, most if not all, containing multiple transmembrane helices. The putative active site of RseP proteases is normally located within the hydrophobic plane of the membrane and contains a consensus sequence motif HExxH, in which the two histidine residues are thought to coordinate a zinc atom together with a conserved glutamate residue (Feng et al., 2007; Koide et al., 2007; Rawson, 2013). Our results indicate that the active site of RseP is somehow involved in the sensitivity to the bacteriocins. This is based on the observation that replacing of the conserved residues with alanine residues in *E. faecalis* RseP made the cloned cells less sensitive to EntEJ97 and EntK1 especially when all three conserved residues were replaced. This finding is different from other known bacteriocin receptor systems. Substitution mutations of conserved active site residues in LsrS – Zn-dependent membrane protein, serving as receptor for the two-peptide lantibiotic bacteriocin Smb, had no effect on cells sensitivity to the bacteriocin (Biswas and Biswas, 2014). Similarly, the man-PTS membrane-located components IIC and IID can serve as a receptor for pediocin-like bacteriocins even without its cytosolic component (IIAB) which is needed for functional sugar transport (Diep et al., 2007). How the active site of RseP involved in the sensitivity to EntK1/EntEJ95 is still elusive. The structure of RseP homologous protein from archaebacterial species *Methanocaldococcus jannaschii* showed that it is quite dynamic during the proteolytic reaction. The protein displays structural changes including the movement of the transmembrane helices to bring the substrate (a peptide or part of a protein) into the active site and to release the digested product after proteolysis (Feng et al., 2007). It is tempting to speculate that site-directed mutations in the active site of RseP might have altered its 3D-structure, making it less accessible for the bacteriocins. Other possibilities might also exist. For example, these mutations could somehow create a less stable or incorrectly folded RseP protein that was removed from cells by chaperone proteases.

The study of EntK1 and EntEJ97 inhibition spectrum showed an interesting detail – EntK1 was less active and had generally narrower antibacterial spectrum than EntEJ97. However, against *E. faecium* strains EntK1 was much more potent (i.e., lower MIC values) than EntEJ97. The latter was equally active against *E. faecalis* and *E. faecium* (**Table 4**). Since EntK1 is produced by *E. faecium* and EntEJ97 by *E. faecalis*, this difference is in line with the general characteristic of bacteriocins, namely that they are most active against species closely related to the producers. This narrow-spectrum activity is not well understood in terms of mechanisms, but likely has an ecological meaning, e.g, giving the bacteriocin producer an advantage in competition for nutrients or habitats over its closest competitors. However, multiple sequence alignment analysis (using Clustal Omega) of many RseP (30 from *E. faecalis* and 30 from *E. faecium*) did not show any pronounced differences between the two RseP groups (data not shown). Thus, what properties of RseP that define the different levels of sensitivity toward EntEJ97 and EntK1 awaits further investigation.

During the past few decades, enterococci have emerged as important healthcare-associated pathogens due to their resistance to antibiotics and environmental stress factors (Arias and Murray, 2012). The rapid spread of *E. faecium* with resistance to vancomycin (VRE), ampicillin and high-levels of aminoglycosides is of particular concern (Agudelo Higuita and Huycke, 2014). *E. faecium* is now a nosocomial pathogen as common as *E. faecalis* (Arias and Murray, 2012). Recently RseP was shown to be a major virulence determinant in enterococcal rabbit endocarditis model (Frank et al., 2012). The importance of RseP in virulence is most likely due to its role in stress response (Varahan et al., 2013). Therefore, EntK1 and EntEJ97, both targeting enterococcal RseP, can be used in combination with stress factors to effectively kill antibiotic-resistant enterococci including VRE.

## Author Contributions

KO: NMR of EntK1, heterologous expression of rseP, MIC assays, and paper writing. PK: NMR of EntK1. DS: heterologous expression of rseP. MJ: rseP mutant characterization, MIC assays. TA-P: paper writing. IN: paper writing. DD: project leadership and paper writing.

## Conflict of Interest Statement

The authors declare that the research was conducted in the absence of any commercial or financial relationships that could be construed as a potential conflict of interest.
