# Supplementary material for: The Leaderless Bacteriocin Enterocin K1 Is Highly Potent against Enterococcus faecium: A Study on Structure, Target Spectrum and Receptor
Source: Front Microbiol. 2017 May 3;8:774. doi: 10.3389/fmicb.2017.00774 (PMC5413573; doi:10.3389/fmicb.2017.00774)
Supplement: Supplementary file 1 [file Image_1.PDF]

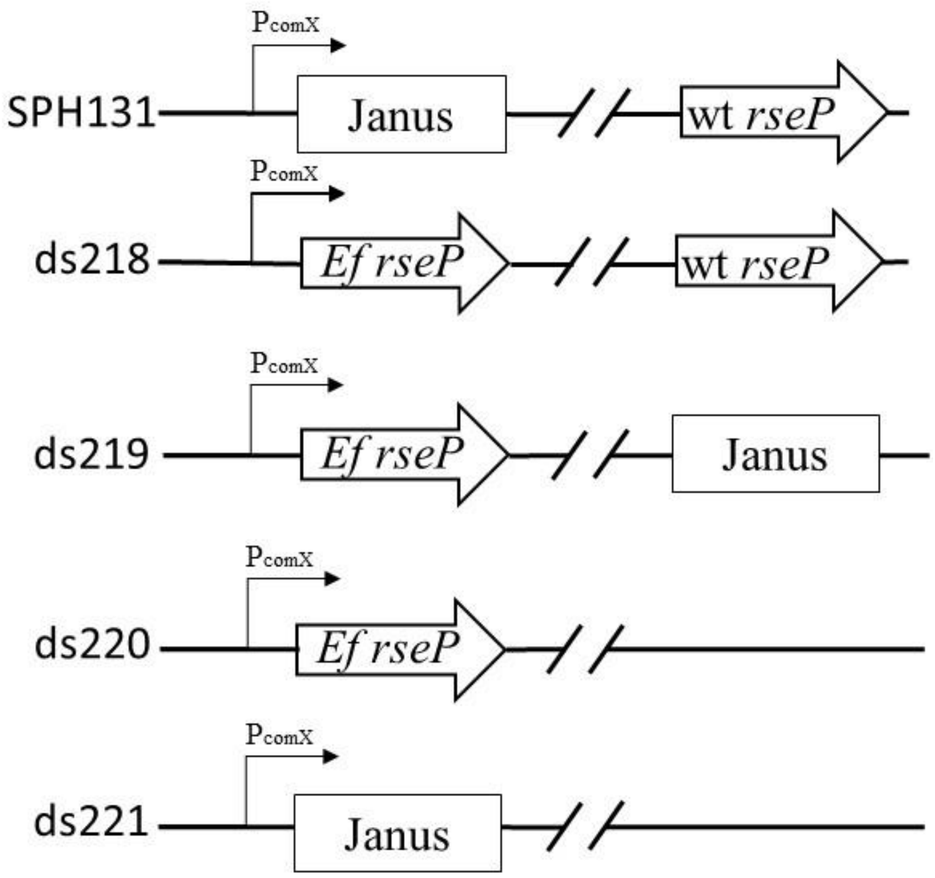

Supplementary FIG 1

Construction of *S. pneumoniae* transformants and mutants of *rseP*. Scheme of the experiment.
