# Supplementary figures and images for: The Leaderless Bacteriocin Enterocin K1 Is Highly Potent against Enterococcus faecium: A Study on Structure, Target Spectrum and Receptor
Source: Front Microbiol. 2017 May 3;8:774. doi: 10.3389/fmicb.2017.00774 (PMC5413573; doi:10.3389/fmicb.2017.00774)

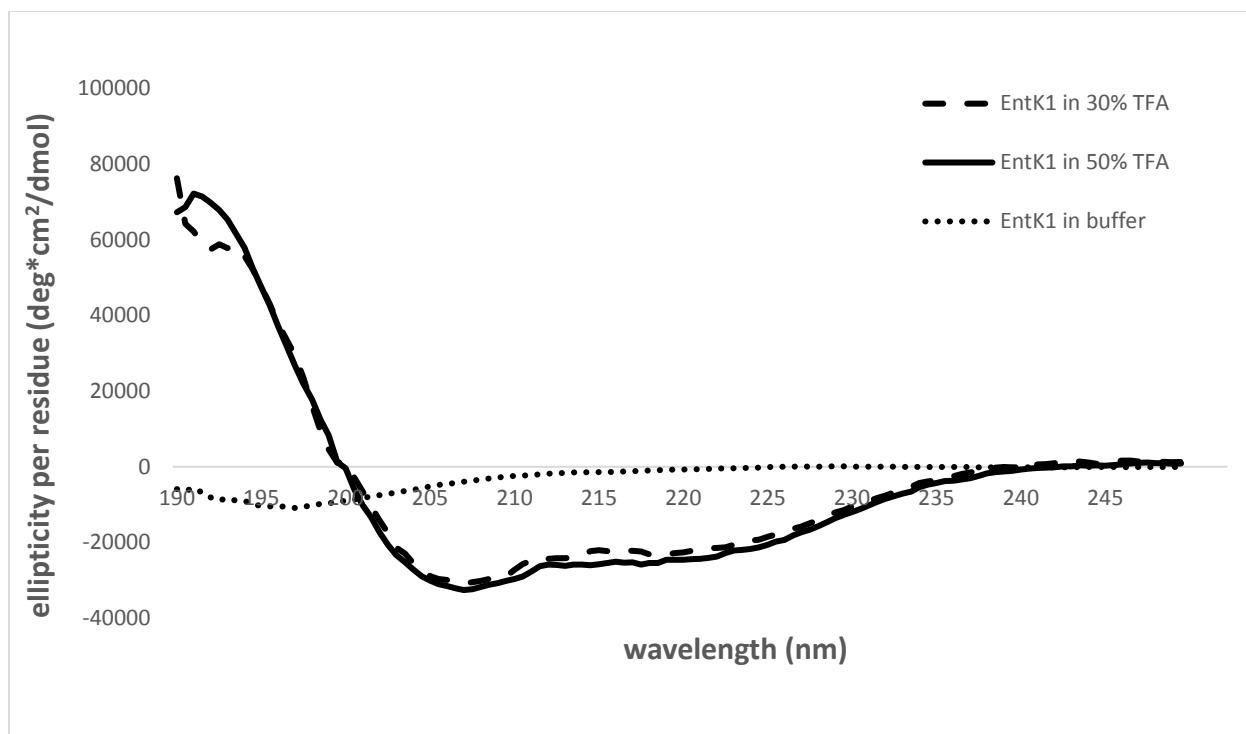

**Supplementary FIG 2.**

CD spectra of EntK1 in buffer, 30% and 50% TFE.

Supplement: Supplementary file 2 [file Image_2.PDF]

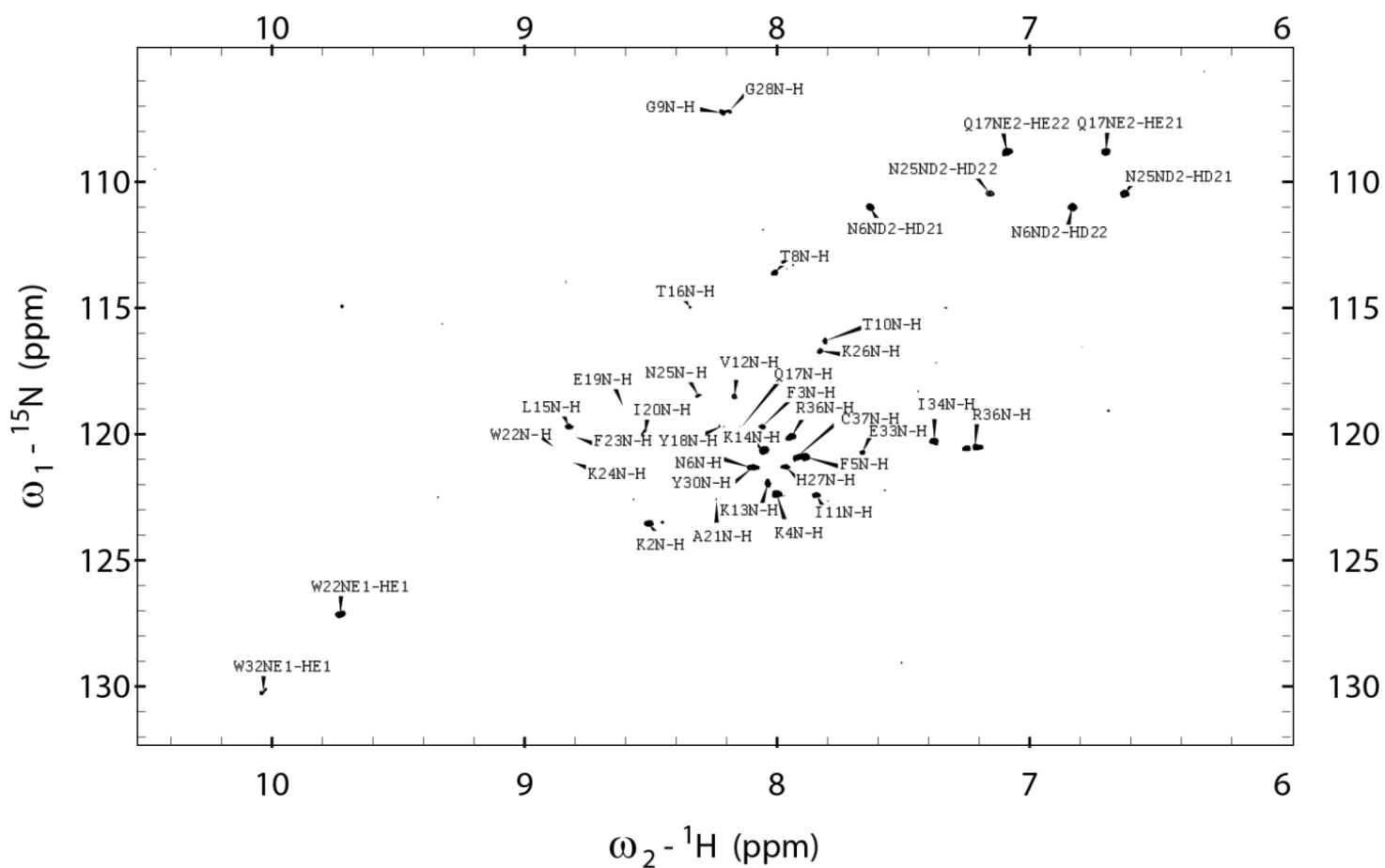

**Supplementary FIG 3.**

${}^{15}\text{N}$  HSQC spectrum of EntK1 in 50% TFE with chemical shift assignments.

Supplement: Supplementary file 3 [file Image_3.PDF]
